# Identification of *TXNIP, FTCD,* and *HAGH* as Key Genes in a Cancer Stem Cell-driven Prognostic Model for Hepatocellular Carcinoma

**DOI:** 10.2174/0118715303482356260608112834

**Published:** 2026-06-22

**Authors:** Xieling Yin, Yuan Zhou, Shi Chen, Chunyang Ma, Hongfei Wu, Hui Cao, Wei Shi, Chi Liang

**Affiliations:** 1 Department of Hepatobiliary Surgery, Tumor Hospital Affiliated To Nantong University, Nantong Tumor Hospital, Nantong, 226006, China;; 2 Department of General Surgery, Huashan Hospital, Fudan University, Shanghai, 200040, China;; 3 Department of General Surgery, Suqian First People’s Hospital, Suqian, 223800, China

**Keywords:** Hepatocellular carcinoma, cancer stem cells, immune microenvironment, single-cell RNA sequencing, hdWGCNA, prognostic model

## Abstract

**Introduction:**

This study developed a prognostic model for hepatocellular carcinoma (HCC) based on cancer stem cell (CSC)-related modules identified by high-dimensional WGCNA (hdWGCNA).

**Methods:**

The scRNA-seq data were filtered, dimensionally reduced, and clustered for downstream analysis. CSC-related modules were identified by hdWGCNA. Based on the TCGA liver hepatocellular carcinoma (LIHC) data, we developed a prognostic RiskScore model using univariate Cox, LASSO, and stepwise regression analyses and validated in the ICGC-LIRI-JP dataset. Correlations between the model and the tumor immune microenvironment (TME) and drug sensitivity were analyzed.

**Results:**

Nine cell subpopulations, including CSCs, were identified and were significantly enriched in tumors. hdWGCNA identified six CSC-related key modules. The prognostic RiskScore model, developed based on TXNIP, FTCD, and HAGH, exhibited an AUC > 0.6 in both cohorts. A high RiskScore was correlated with an immunosuppressive TME characterized by downregulated neutrophils, NK cells, and eosinophils, and higher sensitivity to chemotherapeutic agents such as Pyrimethamine and Vinorelbine.

**Discussion:**

This study identified CSC-related modules associated with HCC prognosis, TME, and drug response, providing a potential mechanistic basis for the clinical application of the model.

**Conclusion:**

Potential prognostic and targeted therapy biomarkers were identified. The CSC-related module-based prognostic model may offer a new tool for personalized HCC treatment.

## 1 INTRODUCTION

As a major cause of global cancer-related mortality, liver cancer shows rising death rates [1-3]. Primary hepatic squamous cell carcinoma (SCC) is extremely rare, representing a rare and clinically challenging pathological subtype with a low incidence and poor prognosis. At present, surgical intervention is the primary HCC treatment option. The 5-year survival rate of patients undergoing surgical resection exceeds 60%; however, more than 70% of these patients relapse within 5 years post-surgery [4]. Radiation therapy is also one of the important therapeutic modalities for HCC. Intra- and peritumoral radiomics based on ^18^F-FDG PET/CT plays a vital role in predicting the prognosis of HCC. This approach substantially improves prognostic prediction in liver cancer and is significantly more effective than conventional single imaging and routine metabolic parameters [5]. In addition, liver tumors can be treated with ablative radiation therapy (RT), which also contributes greatly to the management of hepatocolic fistula after radiation lobectomy. Toll-like receptors (TLRs) play a pivotal role in regulating liver regeneration following partial hepatectomy by modulating inflammation and hepatocyte proliferation [6]. Despite the availability of diverse therapeutic options for liver cancer, its age-standardized incidence and mortality vary widely worldwide, and the overall prognosis remains poor, with a 5-year overall survival below 20% [7], underscoring an urgent demand for novel, precise biomarkers and personalized therapies.

Cancer stem cells (CSCs), also known as tumor-initiating cells, are a tumor cell subpopulation characterized by quiescence, pluripotency, and self-renewal. These cells were initially identified in hematological malignancies and have been confirmed in solid tumors [8]. Due to their self-renewal, differentiation potential, and chemoresistance, CSCs are considered key drivers of tumor initiation, recurrence, and metastasis; however, their dynamic behaviors and regulatory mechanisms remain incompletely elucidated. In HCC, studies have reported the crucial biological functions of liver cancer stem cells (LSCs) in tumor dissemination and metastasis [9]. Since CSCs play pivotal roles in HCC initiation, progression, metastasis, and therapy resistance [10], in-depth exploration of their biological characteristics and regulatory mechanisms may help facilitate the development of novel therapeutic strategies and improve clinical outcomes for HCC patients.

Existing prognostic models for CSCs-related HCC are often limited either by the insufficient depth of bulk RNA sequencing alone [11] or by the narrow scope of conventional screening strategies [12]. In this study, we introduced the hdWGCNA algorithm to achieve a refined delineation of cancer stem cell functional modules at single-cell resolution. Furthermore, an integrated nomogram was constructed on this basis to overcome the limitations of previous models. Through multivariate regression and calibration validation, we established a RiskScore, which serves as a key prognostic indicator independent of the AJCC staging system and can significantly enhance the accuracy of prognostic prediction in HCC. This approach is expected to advance HCC treatment toward personalized and precision medicine.

## 2 MATERIALS AND METHODS

### 2.1 Acquisition of Analytical Data

FPKM format of HCC and clinical data were obtained from the TCGA-liver hepatocellular carcinoma (LIHC) database [13]. After excluding samples lacking survival time, survival status, or with survival time ≤ 0 days, a total of 365 HCC samples were ultimately retained. To ensure data quality, only protein-coding genes were included for subsequent analyses. This curated dataset was designated as the training set for the present study.

The ICGC-LIRI-JP dataset was downloaded from the HCCDB18 database (http://lifeome.net/database/hccdb/home.html). After excluding samples lacking survival time or survival status, and those with survival time ≤0 day, 203 HCC samples were obtained and used as the validation set.

The Gene Expression Omnibus (GEO) database was accessed to obtain GSE149614, a single-cell dataset of HCC. Non-tumor samples and primary tumor samples were selected based on the clinical data. Sequencing libraries were prepared using the 10x Genomics platform and sequenced on the Illumina NovaSeq 6000.

### 2.2 Filtering, Dimensionality Reduction, and Clustering of Single-cell Transcriptome Sequencing Data

A single-cell object was constructed using the CreateSeuratObject function in the Seurat v3.2.3 package [14], and only cells with 200–6500 detected genes and a mitochondrial gene percentage <10% were retained. After normalizing the data using the NormalizeData function, highly variable genes were selected using the FindVariableFeatures function. These identified genes were subsequently scaled using the ScaleData function. Principal component analysis (PCA) was conducted using the RunPCA function, and batch effects were corrected using Harmony [15]. Based on the Harmony-corrected low-dimensional space, dimensionality reduction and visualization were performed using UMAP. Cells were clustered using the FindNeighbors and FindClusters functions. Finally, cell type annotation for each cluster was performed based on both the CellMarker 2.0 database [16] and differential expression analysis results.

### 2.3 Differential Expression Analysis Between Cell Subpopulations

Genes with specifically high expression among different subpopulations were identified using FindAllMarkers (parameters: logfc.threshold = 0.25, min.pct = 0.30, and only.pos = T).

### 2.4 Screening of CSC-related Genes by hdWGCNA

The hdWGCNA package [17] was utilized to read single-cell transcriptomic RDS data. Genes expressed in ≥5% of cells were retained to initialize the WGCNA object. Subsequently, the K-nearest neighbors (KNN) algorithm (k = 25) was used to aggregate cells into metacells to mitigate the sparsity of the single-cell expression matrix. We employed the pickSoftThreshold function to automatically determine the optimal soft-thresholding power. First, the quality-controlled and annotated single-cell Seurat object was imported, and the cancer stem cell subpopulation was extracted for subsequent co-expression network construction. Network topology analysis identified the optimal soft threshold as 12, which was used to construct a gene co-expression network with an approximate scale-free distribution. Subsequently, co-expression modules were classified based on pairwise gene co-expression relationships, yielding a total of 28 modules (M1–M28).

To screen core hub genes within each module, the correlation between each gene and the module eigengene (kME) was calculated. A higher kME value indicates stronger consistency with the module's overall expression pattern and greater connectivity within the module. Instead of applying a fixed kME cutoff, genes were ranked by kME within each module, and the top 15 genes with the highest kME values in each module were selected as candidate hub genes using the GetHubGenes() function. The obtained hub genes were further utilized for screening prognostic genes and developing a RiskScore model.

### 2.5 Functional enrichment analysis

Using the “clusterProfiler” R package [18], Gene Ontology (GO) and Kyoto Encyclopedia of Genes and Genomes (KEGG) enrichment analyses were performed based on the selected hub genes in the module.

### 2.6 Construction and verification of RiskScore models

To construct a prognostic risk model associated with OS in patients with LIHC, candidate hub genes identified by hdWGCNA were combined with the expression profiles and clinical survival data of the TCGA-LIHC cohort. Univariate Cox regression analysis was performed to preliminarily screen candidate hub genes significantly correlated with OS. Subsequently, LASSO-Cox regression analysis was conducted using the R package glmnet [19] to reduce the number of genes and mitigate overfitting. The LASSO-Cox model was set with alpha = 1, and the optimal penalty parameter lambda.min was determined *via* 10-fold cross-validation. Seven genes with non-zero regression coefficients under the lambda.min parameter were retained as candidate prognostic genes and incorporated into a multivariate Cox regression model, followed by stepwise regression analysis using the R step() function, with variable selection based on the Akaike information criterion (AIC). The RiskScore was formulated as follows:


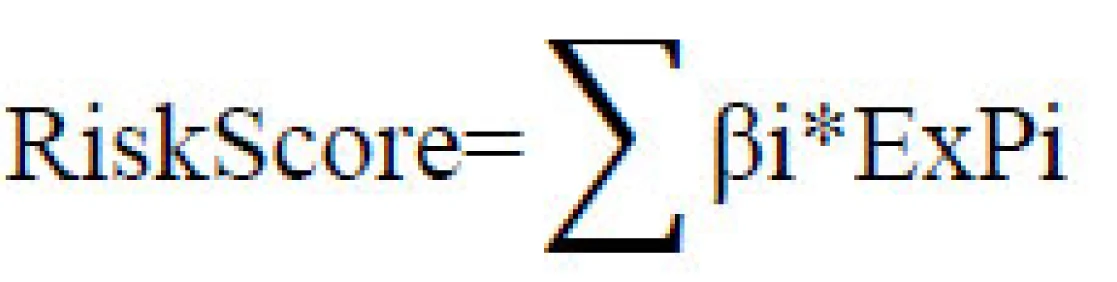


β is the coefficient of the gene, and i is the gene expression.

Using the survminer package [20], the optimal cutoff value for the RiskScore was determined with OS time and status as outcome variables. Patients in the TCGA-LIHC training cohort and the ICGC-LIRI-JP external validation cohort were then divided into high-risk and low-risk groups. Kaplan–Meier (KM) survival analysis combined with the log-rank test was performed to compare OS differences between the two groups. Time-dependent ROC curves were used to evaluate the model’s predictive performance for 1-, 3-, and 5-year OS, and corresponding AUC values were calculated. The ICGC-LIRI-JP cohort was used for external validation to assess the model's generalizability. Whether the RiskScore was an independent prognostic factor was verified by performing univariate and multivariate Cox regression analyses on the RiskScore and clinical variables.

### 2.7 Analysis of a Nomogram

The independent prognostic value of the RiskScore, age, gender, AJCC stage, tumor grade, medication therapy, and radiotherapy was assessed by Cox regression analysis. Univariate Cox regression analysis was performed to screen OS-related variables. Subsequently, statistically significant variables were incorporated into the multivariate Cox regression model to determine whether RiskScore was an independent prognostic factor. The results were presented as hazard ratios (HRs), 95% confidence intervals (95% CIs), and *P* values, and visualized in a forest plot.

A nomogram was constructed based on the significant variables derived from multivariate Cox regression to predict the 1-, 3-, and 5-year OS for patients. Calibration curves were plotted to assess the consistency between predicted outcomes and actual survival status, and decision curve analysis (DCA) was used to evaluate the clinical application value of the nomogram model.

### 2.8 Tumor Immune Microenvironment

First, enrichment scores of 28 types of infiltrating immune cells in the samples were computed using the ssGSEA algorithm in the GSVA package [18]. Subsequently, the MCP-counter package [21] was employed to estimate the relative abundances of 10 immune and stromal cell subsets in the same cohort. Finally, patients were divided into high-risk and low-risk groups based on the RiskScore. To evaluate immune response differences between the risk subgroups, differential expression analysis of common immune checkpoint genes was performed.

### 2.9 Drug Sensitivity

The pRRophetic package [22] was used to predict the sensitivity of the patients in TCGA-LIHC to common chemotherapeutic drugs and to calculate the correlation between IC_50_ and the RiskScore.

### 2.10 Statistical Analysis

Statistical analyses were performed using the R package (version 3.6.0). The Wilcoxon rank-sum test was applied for two-group comparisons of continuous variables; correlations were assessed *via* the Spearman method; survival differences across groups were evaluated with the log-rank test. A *p* < 0.05 was considered statistically significant. This study strictly adhered to the TRIPOD reporting guidelines (Table **S1**).

## 3 RESULTS

### 3.1 Single-cell Atlas of HCC

**Fig. (1):**
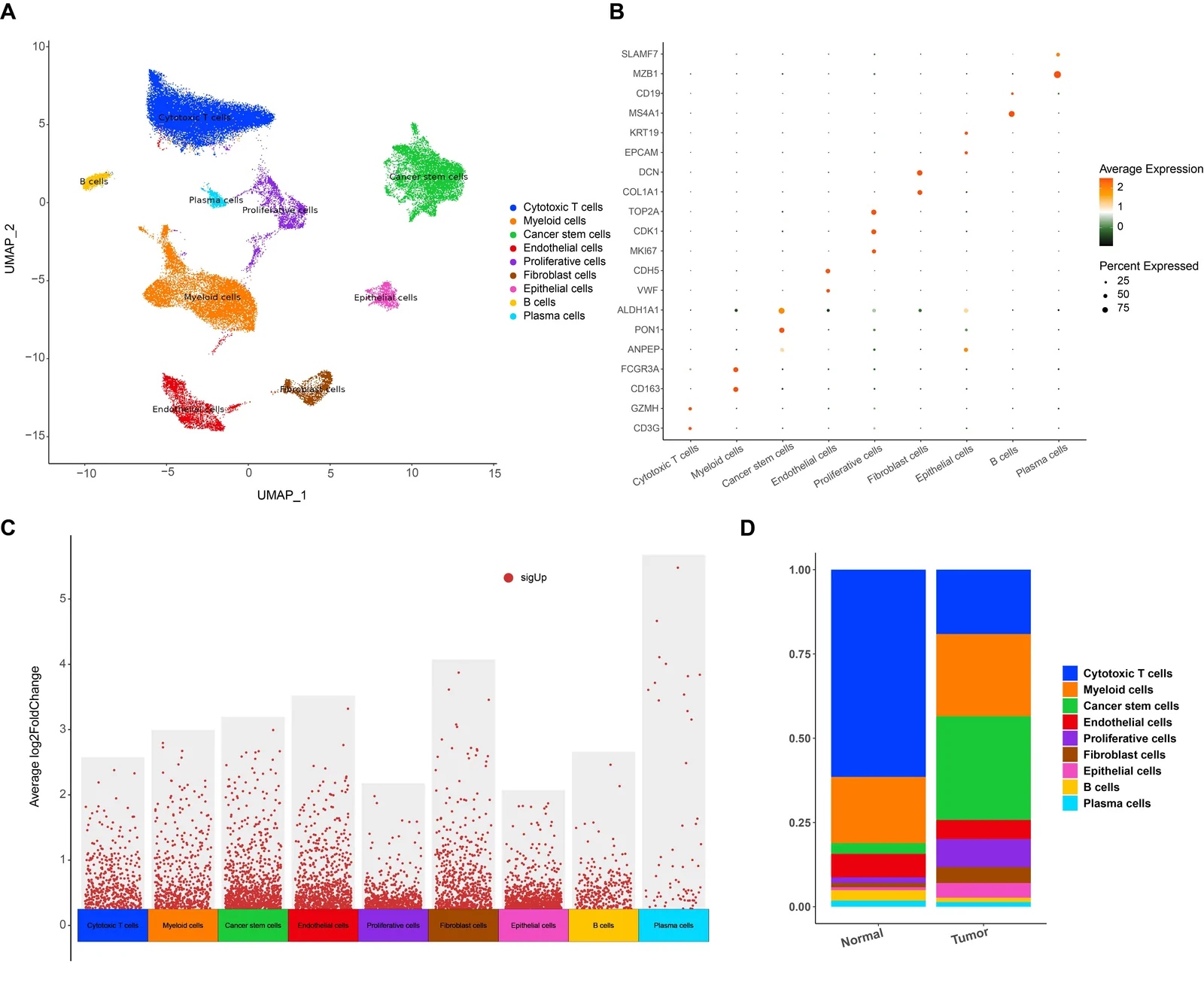
HCC single-cell atlas. (**A**) UMAP dimensionality reduction plot for single-cell clustering and annotation; (**B**) Bubble plot of marker gene expression used for subpopulation annotation; (**C**) Heatmap of differentially expressed genes in each cell subpopulation; (**D**) Relative abundance distribution of each cell subpopulation in tumor and non-tumor groups.

Single-cell transcriptome analysis identified nine cell subpopulations between primary tumor and adjacent non-tumor liver samples, including cytotoxic T cells, myeloid cells, cancer stem cells, endothelial cells, proliferative cells, fibroblast cells, epithelial cells, B cells, and plasma cells (Fig. **1A**). The cell type annotations were further validated by examining the expression of characteristic marker genes. For example, *EPCAM* and *KRT19* were highly expressed in epithelial cells; *CDH5* and *VWF* were highly expressed in endothelial cells; and stemness marker genes such as ALDH1A1 and PON1 were highly expressed in CSCs (Fig. **1B**). Furthermore, DEGs across various cell populations were analyzed (Fig. **1C**), and the proportional changes in tumor and non-tumor cells were compared (Fig. **1D**). These results demonstrated that the relative abundance of CSCs increased most significantly in tumor samples, suggesting that this cell type functions importantly in HCC development.

### 3.2 hdWGCNA Screening for CSC-related Modules

**Fig. (2):**
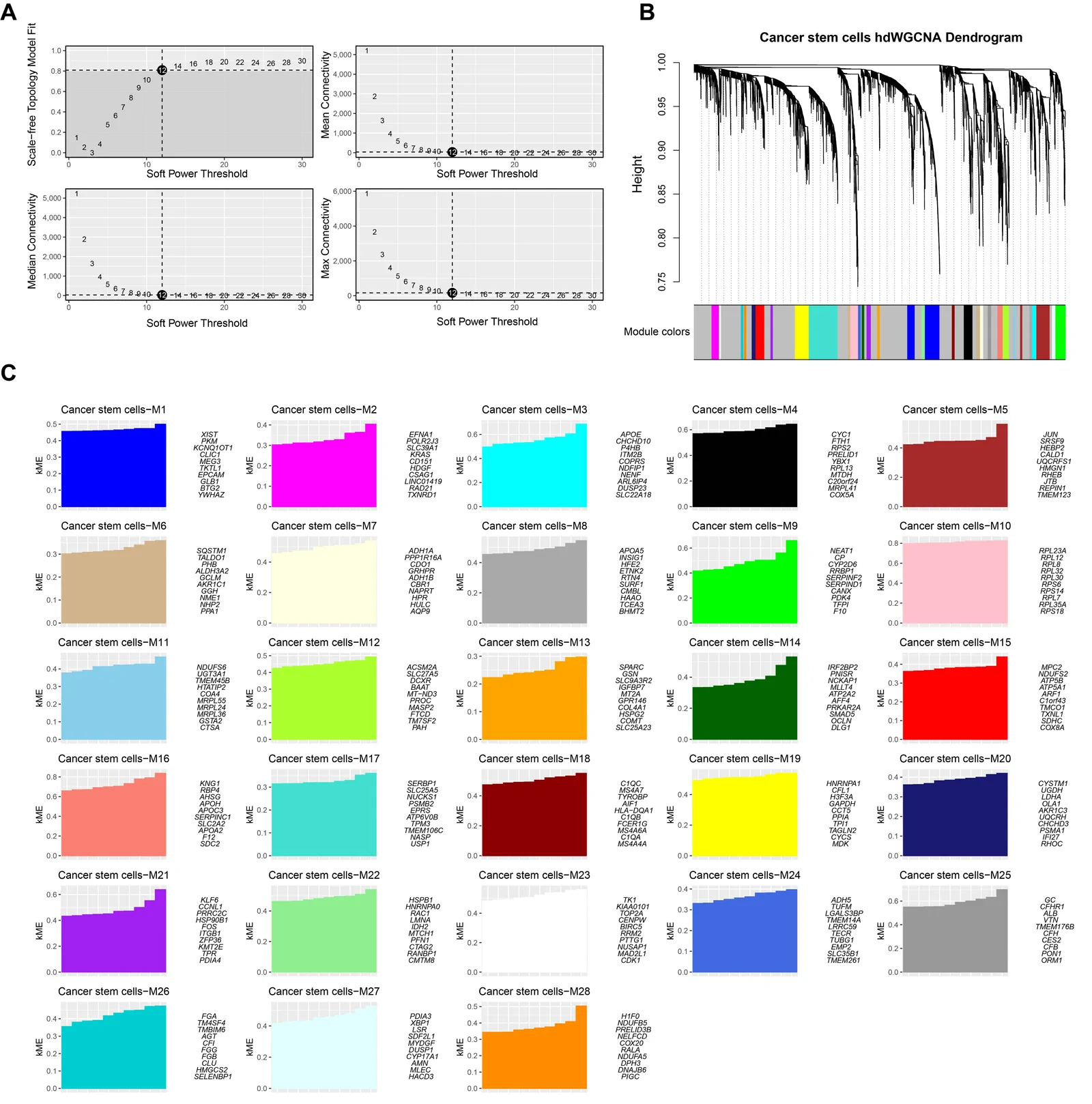
Identification of CSC-related modules using hdWGCNA. (**A**) Soft Threshold Selection, with the optimal soft threshold identified as 12; (**B**) Co-expression Network Dendrogram, with the upper half representing the gene tree where each branch corresponds to a gene, and the lower half indicating the modules associated with the genes; (**C**) Module Assignment, with the y-axis representing the kME values for each gene, and the right side indicating the hub genes of the modules.

With CSC subpopulations as the main subject of analysis, the optimal soft-thresholding power of 12 was determined using pickSoftThreshold (Fig. **2A**) to develop a weighted co-expression network (Fig. **2B**). Based on module eigenvectors (ME) and gene intramodular connectivity, we divided 28 co-expression modules (M1-M28) and selected highly connected genes of each module (Fig. **2C**).

**Fig. (3):**
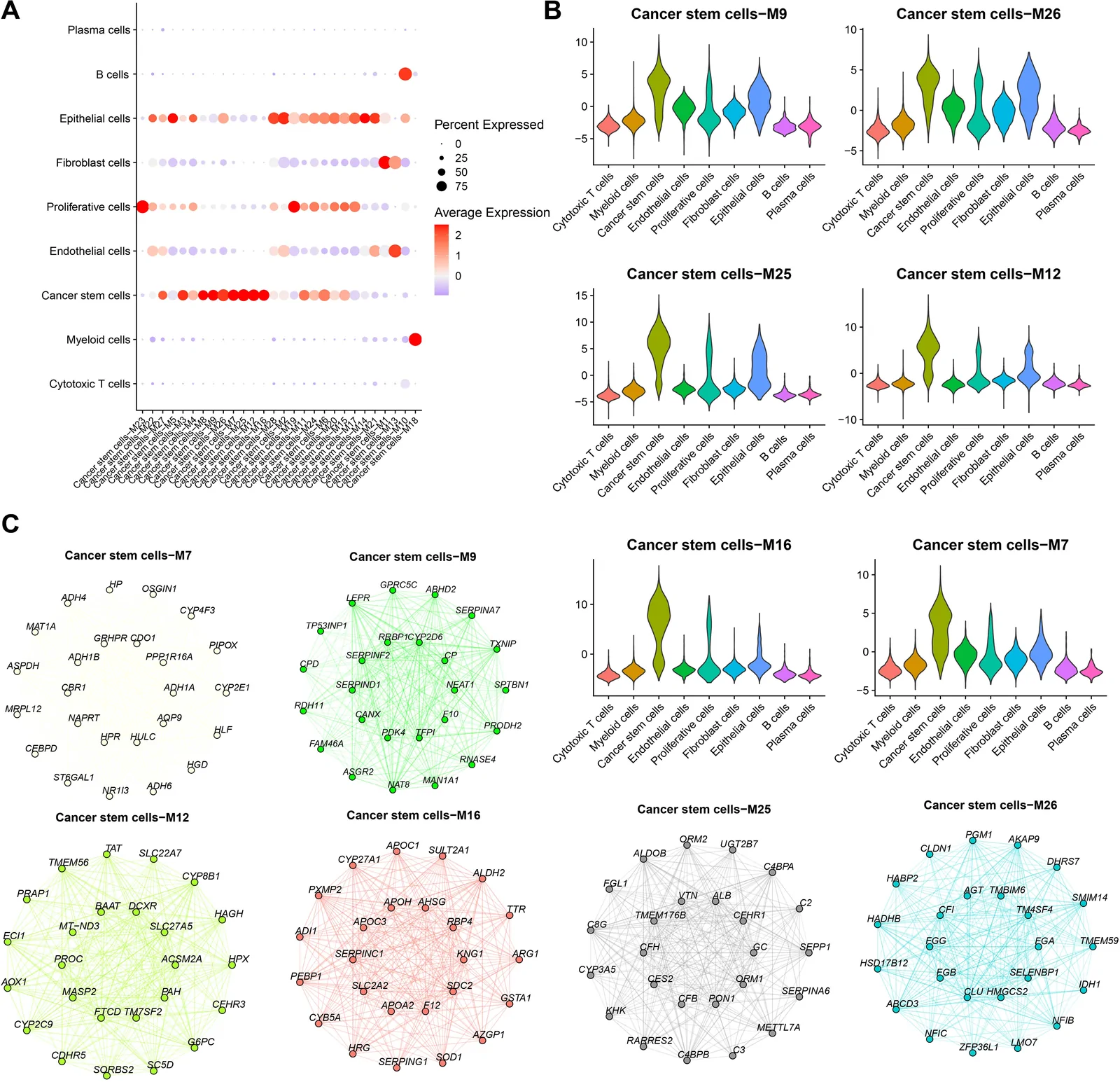
Gene expression and network analysis of key modules. (**A**) Gene expression within the module in different cells, where red and purple represent high and low expressions, and the circle size indicates cell proportion; (**B**) The expression distribution of ME of the six cancer stem cell-related co-expression modules in nine annotated cell types. (**C**) Co-expression network of hub genes in the six cancer stem cell-related co-expression modules, with the inner circle representing key genes and the outer circle representing secondary key genes.

Notably, genes in modules M9, M26, M7, M25, M12, and M16 exhibited a higher proportion and higher expression in CSCs (Figs. **3A** and **3B**). Based on these findings, these six modules were considered key modules, and their hub gene networks were extracted (Fig. **3C**). Among them, the 10 innermost genes were identified as core hub genes, while the 15 outer genes were classified as secondary hub genes.

### 3.3 Pathway Enrichment Analysis of Hub Genes in CSC-associated Co-expression Modules

**Fig. (4):**
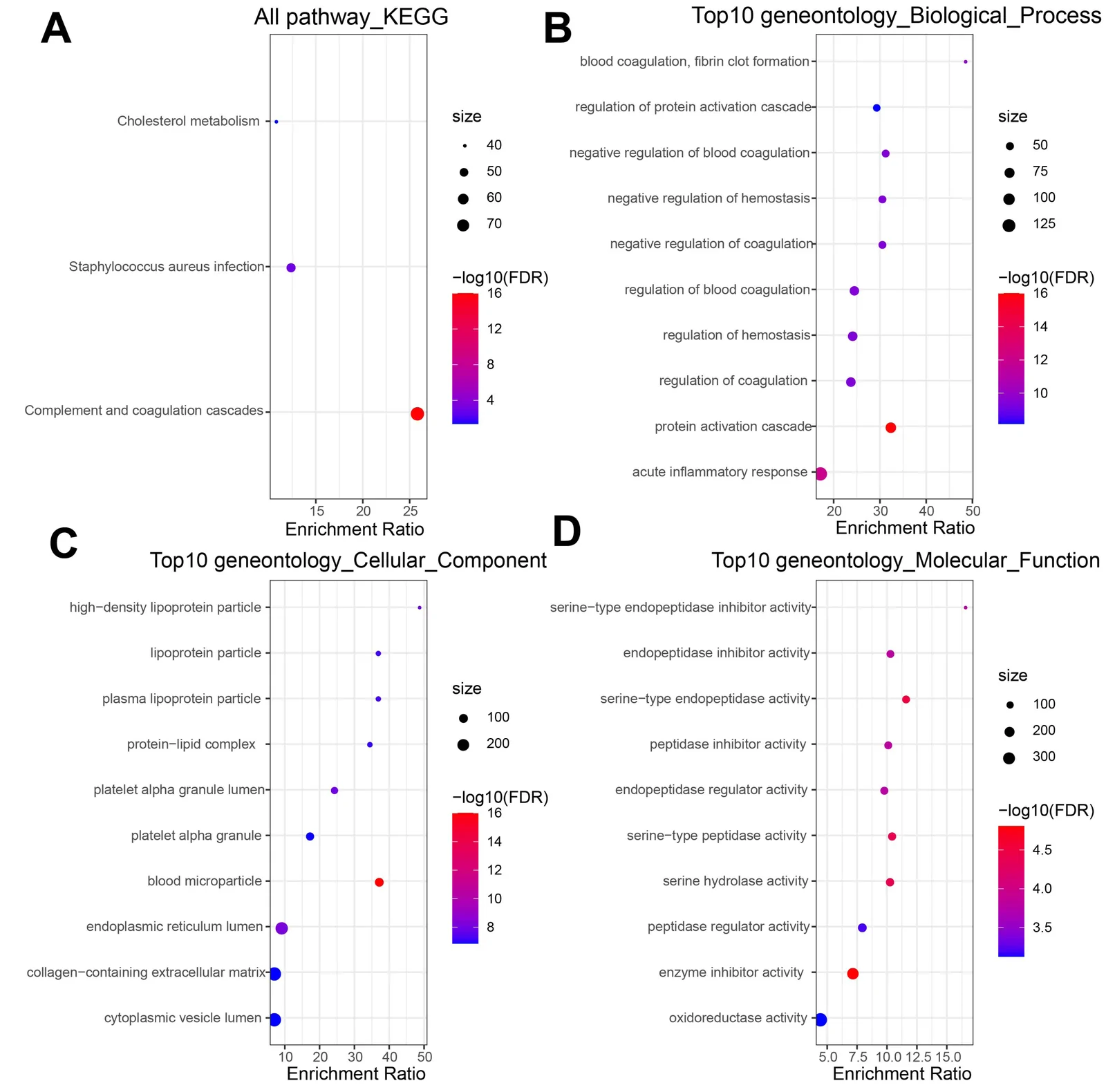
Results of pathway enrichment analysis for hub genes in six cancer stem cell-related co-expression modules. (**A**) KEGG enrichment analysis; (**B**) Bar chart of GO enrichment analysis for BP, where the abscissa represents the number of genes included in each term, and the color represents the significance *p*-value (increasing significance from blue to red); (**C**) Bar chart of GO enrichment analysis for CC; (**D**) Bar chart of GO enrichment analysis for MF.

KEGG enrichment analysis showed that these hub genes were mainly enriched in pathways such as cholesterol metabolism and complement and coagulation cascades (Fig. **4A**). GO enrichment analysis showed that the hub genes were primarily enriched in acute inflammatory response and negative regulation of coagulation in the biological process (BP) term (Fig. **4B**). In the cellular component (CC) term, the enriched pathways mainly included platelet alpha granule and endoplasmic reticulum lumen (Fig. **4C**). In the molecular function (MF) term, the enriched pathways mainly included oxidoreductase activity, serine-type endopeptidase inhibitor activity, and endopeptidase inhibitor activity (Fig. **4D**).

### 3.4 Construction and Verification of a RiskScore

**Fig. (5):**
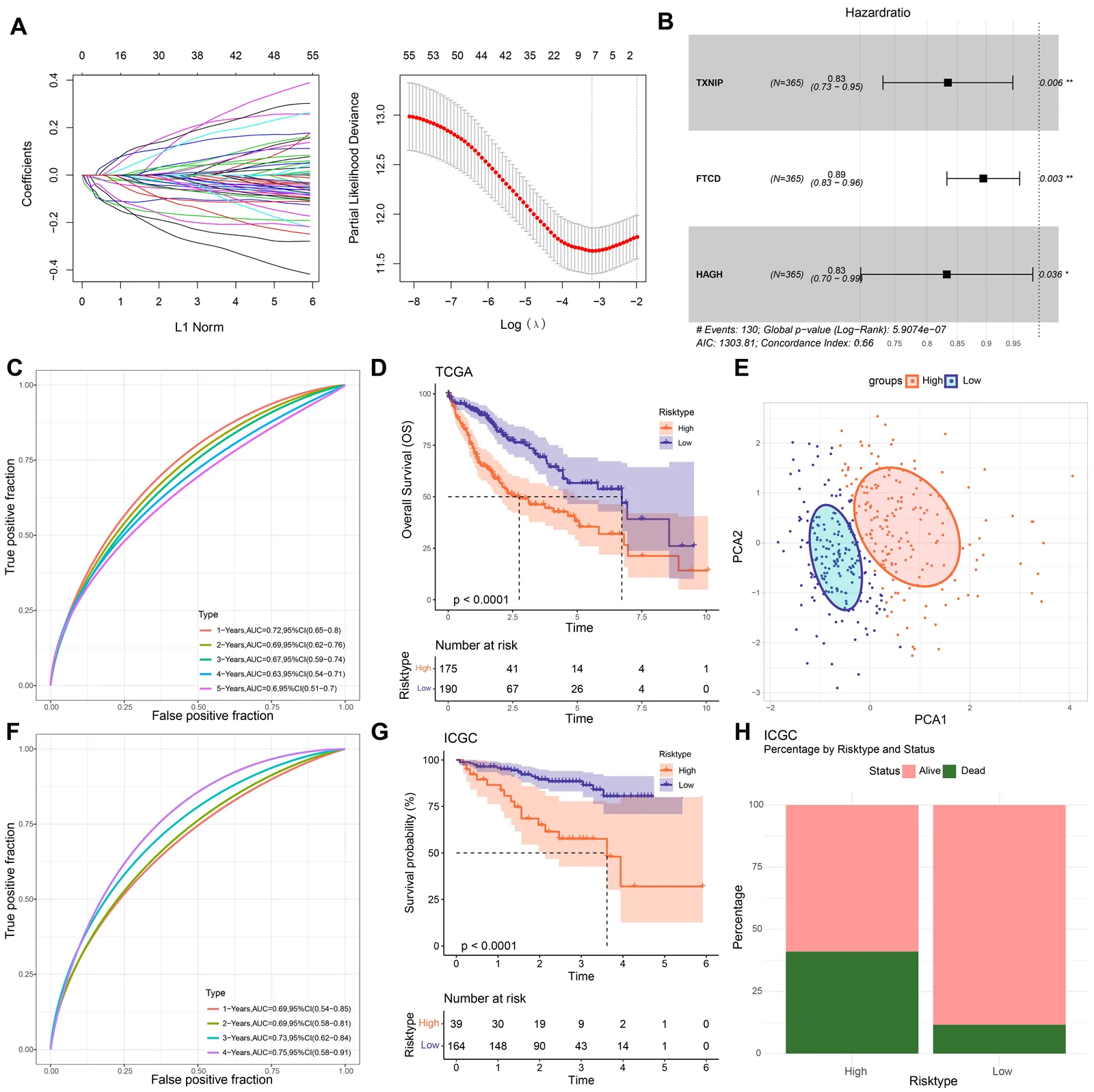
Construction and validation of prognostic RiskScore model. **(A)** LASSO for further narrowing down the range of genes; (**B**) Random forest plot of multivariate factors; (**C-D**) ROC curve and K-M curve of the RiskScore model in the TCGA dataset; (**E**) PCA dimensionality reduction plot based on prognostic genes in the TCGA dataset; (**F-G**) ROC curve and K-M curve of the RiskScore model in the ICGC dataset; (**H**) Differences in survival status between high-risk and low-risk groups of the RiskScore model in the ICGC dataset.* *p*<0.05; ** *p*<0.01.

First, univariate Cox regression analysis screened 57 hub genes closely related to HCC prognosis (*p* < 0.05). Subsequently, LASSO Cox regression analysis combined with 10-fold cross-validation (Fig. **5A**) and stepwise multivariate Cox regression analysis finally selected 3 independent prognosis-related genes for developing a RiskScore model (Fig. **5B**). The model was formulated as follow: RiskScore = (-0.182*TXNIP) + (-0.111*FTCD) + (-0.184*HAGH).

Patients were dichotomized into low- and high-risk groups by the optimal cutoff of the RiskScore. The RiskScore achieved AUC values of 0.72, 0.67, and 0.60 for 1-, 3-, and 5-year OS prediction, respectively (Fig. **5C**), suggesting the reliability of the model. Further K-M survival analysis revealed that the OS was significantly lower in the high-risk group compared to the low-risk group (*p* < 0.0001, Fig. **5D**). As illustrated in Fig. (**5E**), a PCA plot revealed a clear separation between the high-risk and low-risk groups along the PCA1 and PCA2 dimensions, highlighting the substantial transcriptomic differences between the two groups and further supporting the biological relevance and discriminatory power of the RiskScore.

Consistent results were also observed in the ICGC-LIRI-JP dataset (the independent validation cohort). Specifically, patients with a higher RiskScore exhibited a worse prognosis, whereas those with a lower RiskScore exhibited a more favorable prognosis (Fig. **5F** and **5G**). Moreover, more death cases were observed in the high-risk patients in the ICGC-LIRI-JP cohort (Fig. **5H**), thereby further verifying the robustness and generalizability of the model.

After adjustment for clinical factors, including age, gender, AJCC stage, tumor grade, medication therapy, radiotherapy, and the RiskScore, univariate Cox analysis revealed that both AJCC stage and the RiskScore were significantly associated with patients’ OS. Specifically, an elevated AJCC stage indicated an unfavorable prognosis, and an increased RiskScore also significantly raised the death risk (Fig. **S1A**). After further incorporating the significant clinical variables into the multivariate Cox regression model, RiskScore was still selected as an independent risk factor for OS (HR = 2.28, 95% CI: 1.59–3.27, p < 0.001), and AJCC stage also maintained a significant correlation with OS (HR = 1.96, 95% CI: 1.33–2.90, p = 0.001) (Fig. **S1B**).

Based on the results of multivariate Cox analysis, we integrated RiskScore and AJCC stage to construct a nomogram for predicting 1-, 3-, and 5-year OS (Fig. **S1C**). Calibration curves demonstrated that the 1-, 3-, and 5-year survival probabilities predicted by the nomogram were generally in closes agreement with the actual observed outcomes, suggesting a strong calibration capability of the model (Fig. **S1D**). In addition, DCA indicated that within a certain threshold probability range, the nomogram model yielded higher or at least comparable net clinical benefit compared with RiskScore or AJCC stage used alone. This suggests that the nomogram has potential application value in assisting clinical prognostic evaluation (Fig. **S1E**).

Based on the median RiskScore as the cutoff value, patients were stratified into high-risk and low-risk groups. KM survival analysis showed that patients in the high-risk group exhibited a significantly lower OS rate than those in the low-risk group in the TCGA cohort (*p* < 0.0001, Fig. **S2A**). Similarly, markedly lower survival probability was observed in the high-risk group than the low-risk group in the ICGC cohort (*p* = 0.0057, Fig. **S2B**). These findings demonstrated that the model could stably distinguish patients with different prognostic outcomes, indicating high robustness of the model.

### 3.5 RiskScore and Immune Characteristics

**Fig. (6):**
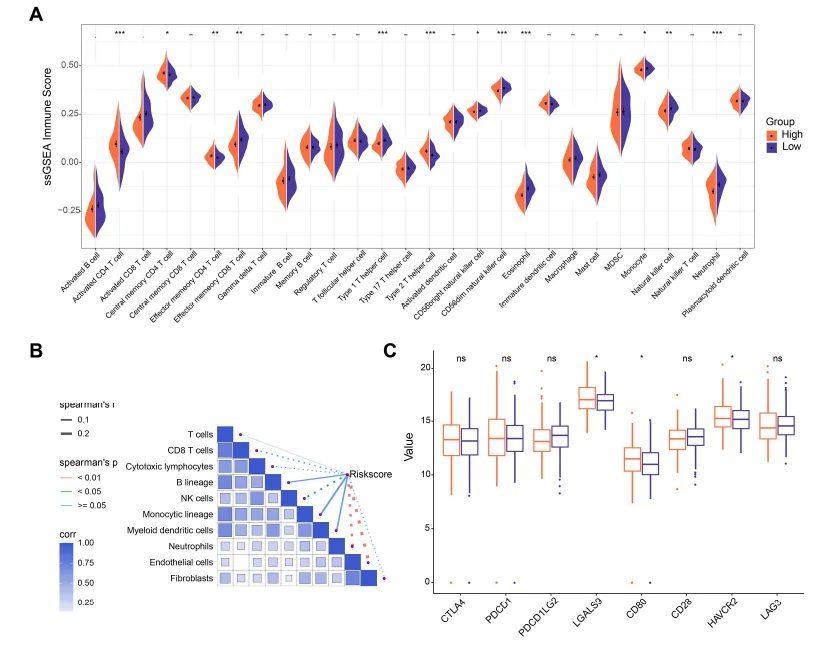
Correlation between immunotherapy response and RiskScore models. (**A**) Expression profiles of 28 ssGSEA immune cell scores grouped by Riskscore; (**B**) Correlation between RiskScores and immune infiltration calculated by the MCPcounter method. Solid and dashed lines indicate positive and negative associations, respectively; (**C**) Immune checkpoint gene expressions in the two risk groups. *** *p*<0.001, ** *p*<0.01; * *p*<0.05.

The results of ssGSEA showed that Th1 cells, NK cells, neutrophils, and eosinophils were significantly downregulated in the high-risk group, while activated CD4 T cells and Type 2 T helper cells were markedly upregulated (Fig. **6A**). The MCP-counter algorithm revealed a significant inverse association between the abundance of NK cells and neutrophils and the RiskScore in the TCGA cohort (Fig. **6B**). Additionally, analysis of immune checkpoint genes showed that the expression of *HAVCR2* and *LGALS9* was significantly higher in the high-risk group, suggesting that an immunosuppressive state may contribute to adverse prognosis (Fig. **6C**).

### 3.6 Drug Sensitivity

**Fig. (7):**
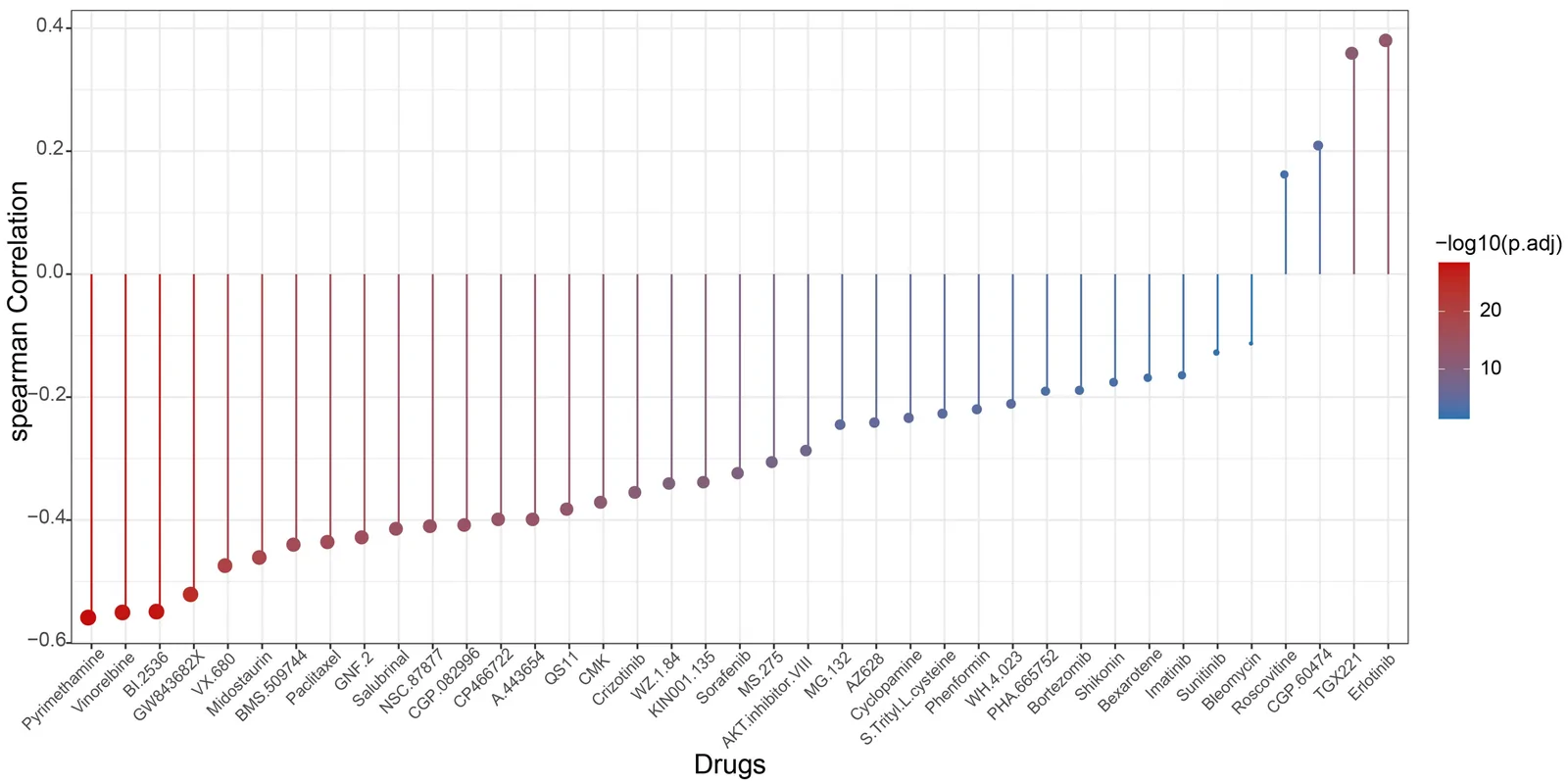
Results of the correlation between Riskscore and drug sensitivity IC_50_.

Drug sensitivity analysis revealed that the IC50 values of several drugs, including Pyrimethamine, Vinorelbine, BI-2536, and GW843682X, were negatively correlated with the RiskScore (Fig. **7**). This suggests that high-risk patients may be more sensitive to these drugs, providing a potential basis for personalized therapies.

## 4 DISCUSSION

HCC is the most common liver cancer subtype worldwide [23-25]. CSCs play a crucial role in HCC by driving tumor growth and are therefore considered crucial targets for diagnosis and treatment of the cancer [26]. In this study, scRNA-seq data were utilized to construct a single-cell atlas of HCC, and a prognostic RiskScore model was developed based on *TXNIP*, *FTCD*, and *HAGH*, demonstrating strong prognostic discrimination ability in both the TCGA training set and the ICGC validation set. Our results validated that the RiskScore was closely related to the immune microenvironment and was associated with the sensitivity to a variety of chemotherapeutic drugs (**e.g.*,* Pyrimethamine and Vinorelbine). This study provided new biomarkers for the prognostic prediction and individualized therapy of HCC.

*TXNIP* is an important protein that regulates intracellular redox status and is involved in cellular glucose and fatty acid metabolism [27]. *TXNIP* inhibits thioredoxin activity, thereby elevating the intracellular oxidative stress level [28]. *TXNIP* plays a dual role in HCC. Under normal conditions, *TXNIP* is lowly expressed in HCC; however, its high expression is significantly associated with a favorable prognosis. Furthermore, *TXNIP* functions as a key mediator of the anti-HCC effects of cordycepin [29]. Within the TME, the expression of *TXNIP* is associated with angiogenesis and inflammatory responses. For example, its expression in hepatic sinusoidal endothelial cells can regulate the production of NO, thereby affecting the progression of HCC. In the alcoholic liver disease (ALD) model, the absence of *TXNIP* exacerbates liver injury, inflammation, and fibrosis, contributing to the occurrence of HCC [30]. The expression of *TXNIP* is regulated by the PIAS3/TGF-β signaling axis, which can also regulate the sensitivity of HCC cells to ferroptosis, providing a potential target for ferroptosis-based therapy of HCC [31]. In conclusion, *TXNIP* plays a complex role in HCC. In the early stage of the disease or under drug induction, it exerts tumor-suppressive effects mainly by inhibiting glycolysis, maintaining cell differentiation, and suppressing stemness. Conversely, in advanced disease stages or specific invasive subtypes, it may promote tumor metastasis through mechanisms such as regulating ROS [32]. *FTCD*, a bifunctional enzyme involved in histidine metabolism, primarily catalyzes reactions in the histidine degradation pathway. *FTCD* is highly expressed in the liver and is closely associated with hepatic metabolic functions [33]. In HCC, *FTCD* is a core gene related to extracellular exosomes, showing the potential to serve as a target for the risk stratification and therapy of HCC. Studies demonstrated that *FTCD* mainly exerts a tumor-suppressive effect in HCC, significantly inhibiting HCC proliferation both *in vitro* and *in vivo* [34]. Additionally, lowly expressed *FTCD* is predictive of worse outcomes of HCC [35]. *Glo2*, encoded by the *HAGH* gene, plays multiple roles in cancer through the regulation of cellular detoxification, regulation of redox balance, and metabolic adaptation, all of which are crucial for the survival and progression of tumor cells [36]. The upregulation of *Glo2* is an adaptive mechanism for tumors to cope with high metabolic stress [37]. Studies on the role of *HAGH* in HCC are limited, and further investigation is needed in the future. In summary, these three genes may affect the occurrence and development of HCC from multiple dimensions, showing the potential to be further studied as indicators for HCC treatment and prognosis.

This study revealed marked differences in the TME between the two risk groups. Th1 cells, a subset of CD4 T helper cells, are primarily involved in cell-mediated immune response *via* the secretion of cytokines such as IFN-γ, IL-2, and TNF-β [38]. These cytokines can activate cytotoxic T lymphocytes (CTLs) and NK cells, thereby enhancing the ability to kill tumor cells. IFN-γ secreted by Th1 cells promotes the presentation of tumor antigens by DCs, thereby activating CTLs and inducing their differentiation into effector CTLs to strengthen the killing effect on tumor cells [39]. Neutrophils promote the progression of HCC through inhibiting anti-tumor immune responses, directly enhancing tumor cell proliferation, and promoting angiogenesis and metastasis [40]. Activated CD4 T cells not only activate cytotoxic CD8 T cells through the secretion of cytokines (*e.g.*, IFN-γ and TNF-α), but also through directly participating in anti-tumor immune responses [41]. In general, these immune cells influence the occurrence and progression of HCC by regulating immune infiltration within tumors.

In this study, we found that the IC_50_ of chemotherapeutic drugs such as Pyrimethamine, Vinorelbine, BI-2536, and GW843682X were significantly negatively correlated with the RiskScores. Among them, Pyrimethamine, a drug commonly used in the treatment of malaria and other protozoal infections, possesses potential anti-tumor activity in various tumors, including HCC [42]. Further studies showed that in different cancer models, Pyrimethamine targets and inhibits a variety of oncoproteins, including STAT3, nuclear factor-κB (NF-κB), DX2, mitogen-activated protein kinase (MAPK), dihydrofolate reductase (DHFR), thymidine phosphorylase, and telomerase [43]. Also, Pyrimethamine exerts a synergistic effect with other anticancer drugs, such as temozolomide, jointly inducing tumor cell apoptosis [43]. Vinorelbine, a semi-synthetic vinca alkaloid, inhibits microtubule polymerization, thereby arresting the mitotic process of cancer cells. Studies confirmed that vinorelbine, in combination with sorafenib, notably improves the treatment outcome of HCC [39]. Additionally, experiments have demonstrated that the combination of vinorelbine with radiotherapy can significantly inhibit tumor growth, showing therapeutic efficacy significantly better than using radiotherapy alone or vinorelbine monotherapy. Furthermore, vinorelbine can increase the sensitivity of liver cancer tumors to standard radiotherapy without increasing treatment-related toxicity [44]. BI.2536 is a small-molecule inhibitor that specifically acts on PLK1, an enzyme that functions crucially in cell mitosis, and its expression is abnormally elevated in various types of cancers. Currently, the specific role and efficacy of BI.2536 in HCC remain to be studied. In summary, these drugs all exhibit a certain potential to inhibit the growth of HCC tumors and induce cancer cell apoptosis; however, whether they can effectively inhibit therapeutic resistance remains to be verified.

In recent years, rapid technological advances have greatly transformed medical practice, among which the Internet of Things (IoT) stands out as one of the most revolutionary breakthroughs. In the treatment of various cancers, including HCC, IoT enables real-time monitoring of patients’ physical conditions, facilitates timely adjustment of therapeutic regimens, and improves postoperative safety management. In addition, artificial intelligence (AI) and image processing technologies exhibit great potential in HCC management. By integrating imaging, clinical, pathological, and molecular data, they support early screening of high-risk populations, accurate lesion diagnosis, optimization of treatment strategies, and prediction of recurrence and prognosis throughout the clinical course of HCC [45]. Three-dimensional (3D) printing assists preoperative planning, doctor–patient communication, and device customization for HCC by constructing high-precision liver models. The prognostic model established in this study provides a potential foundation for clinical application. Future research should further integrate the model with emerging medical technologies to improve its accuracy and practicality.

In the present study, a prognostic prediction model for HCC was constructed based on single-cell transcriptome data and hdWGCNA; however, some limitations should be equally acknowledged. First, the study relied solely on data from the TCGA and ICGC databases, with a relatively limited sample size. This may affect the generalizability and accuracy of the model and restrict its predictive value for long-term prognosis. Second, the model was built only based on gene expression data and was not comprehensively evaluated in combination with other clinical indicators (*e.g.*, imaging features), which may limit its predictive performance. Finally, the drug sensitivity analysis was restricted to selected chemotherapeutic agents and did not include targeted therapy or immunotherapy drugs, thereby limiting the comprehensive guidance for individualized treatment strategies. Future studies are encouraged to expand the sample size, integrate multi-omics data, and validate the clinical utility of the model.

## CONCLUSION

To conclude, this study developed a prognostic model for HCC based on single-cell transcriptome data and identified nine cell subpopulations, including CSCs. The tumor group showed a significantly increased abundance of CSCs. hdWGCNA classified six co-expression modules closely related to CSCs, and their core genes were enriched in pathways such as cholesterol metabolism. A prognostic RiskScore model was further constructed based on TXNIP, FTCD, and HAGH, showing strong prognostic discrimination ability in both the TCGA training set and the ICGC validation set. Meanwhile, the RiskScore was closely related to the immune microenvironment. The high-risk group exhibited a more significant immunosuppressive state and was associated with sensitivity to various chemotherapeutic drugs (*e.g.*, pyrimethamine and vinorelbine). This study provides new biomarkers and a theoretical basis for personalized therapy for HCC and its prognosis.

## Data Availability

All relevant data supporting the findings of this study have been deposited in the Gene Expression Omnibus (GEO) database with the accession number GSE149614 (https://www.ncbi.nlm.nih.gov/geo/query/acc.cgi?acc=GSE149614).

## LISF OF ABBREVIATIONS

ALD: Alcoholic Liver Disease
AUC: Area Under the Curve
BP: Biological Processes
CC: Cellular Components
CTLs: Cytotoxic T Lymphocytes
CSCs: Cancer Stem Cells
DCs: Dendritic Cells
DHFR: Dihydrofolate Reductase
EMT: Epithelial-Mesenchymal Transition
GEO: Gene Expression Omnibus
GO: Gene Ontology
Glo2: Glyoxalase 2
HCC: Hepatocellular Carcinoma
HBV: Hepatitis B Virus
HCV: Hepatitis C Virus
hdWGCNA: High-Dimensional Weighted Gene Co-Expression Network Analysis
Hub Genes: Highly Connected Genes
IC50: Half-Maximal Inhibitory Concentration
ICGC-LIRI-JP: International Cancer Genome Consortium - Liver Cancer - Japan
kME: Module Eigengene Connectivity
KEGG: Kyoto Encyclopedia of Genes and Genomes
KNN: K-Nearest Neighbors
K-M: Kaplan-Meier
LSCs: Liver Cancer Stem Cells
LASSO: Least Absolute Shrinkage and Selection Operator
ME: Module Eigenvectors
MAPK: Mitogen-Activated Protein Kinase
MF: Molecular Functions
NAFLD: Non-Alcoholic Fatty Liver Disease
NO: Nitric Oxide
NF-κB: Nuclear Factor-κB
NK: Natural Killer
OS: Overall Survival
PCA: Principal Component Analysis
PIAS3: Protein Inhibitor of Activated STAT3
PLK1: Polo-Like Kinase 1
ROC: Receiver Operating Characteristic
ROS: Reactive Oxygen Species
scRNA-seq: Single-Cell RNA Sequencing
ssGSEA: Single-Sample Gene Set Enrichment Analysis
STAT3: Signal Transducer and Activator of Transcription 3
TCGA-LIHC: The Cancer Genome Atlas-Liver Hepatocellular Carcinoma
TGF: Transforming Growth Factor
Th1: Type 1 T Helper
OS: Overall Survival
HR: Hazard Ratio
CI: Confidence Interval
DCA: Decision Curve Analysis
SCC: Squamous Cell Carcinoma
RT: Radiation Therapy
TLR: Toll-Like Receptor
IoT: Internet of Things
AI: Artificial Intelligence
3D: Three-Dimensional
