# Supplementary material for: Identification of TXNIP, FTCD, and HAGH as Key Genes in a Cancer Stem Cell-driven Prognostic Model for Hepatocellular Carcinoma
Source: Endocr Metab Immune Disord Drug Targets. 2026 Jun 22;26:E18715303482356. doi: 10.2174/0118715303482356260608112834 (PMC13598741; doi:10.2174/0118715303482356260608112834)
Supplement: Supplementary file 1 [file EMIDDT-26-E18715303482356_SD1.pdf]

Supplementary Material

Identification of *TXNIP*, *FTCD*, and *HAGH* as Key Genes in a Cancer Stem Cell-Driven Prognostic Model for Hepatocellular Carcinoma

Xieling Yin[1,#], Yuan Zhou[1,#], Shi Chen[1], Chunyang Ma[1], Hongfei Wu[1], Hui Cao[1], Wei Shi[2,*] and Chi Liang[3,*]

[1]Department of Hepatobiliary Surgery, Tumor Hospital Affiliated To Nantong University, Nantong Tumor Hospital, Nantong, 226006, China; [2]Department of General Surgery, Huashan Hospital, Fudan University, Shanghai, 200040, China; [3]Department of General Surgery, Suqian First People's Hospital, Suqian, 223800, China

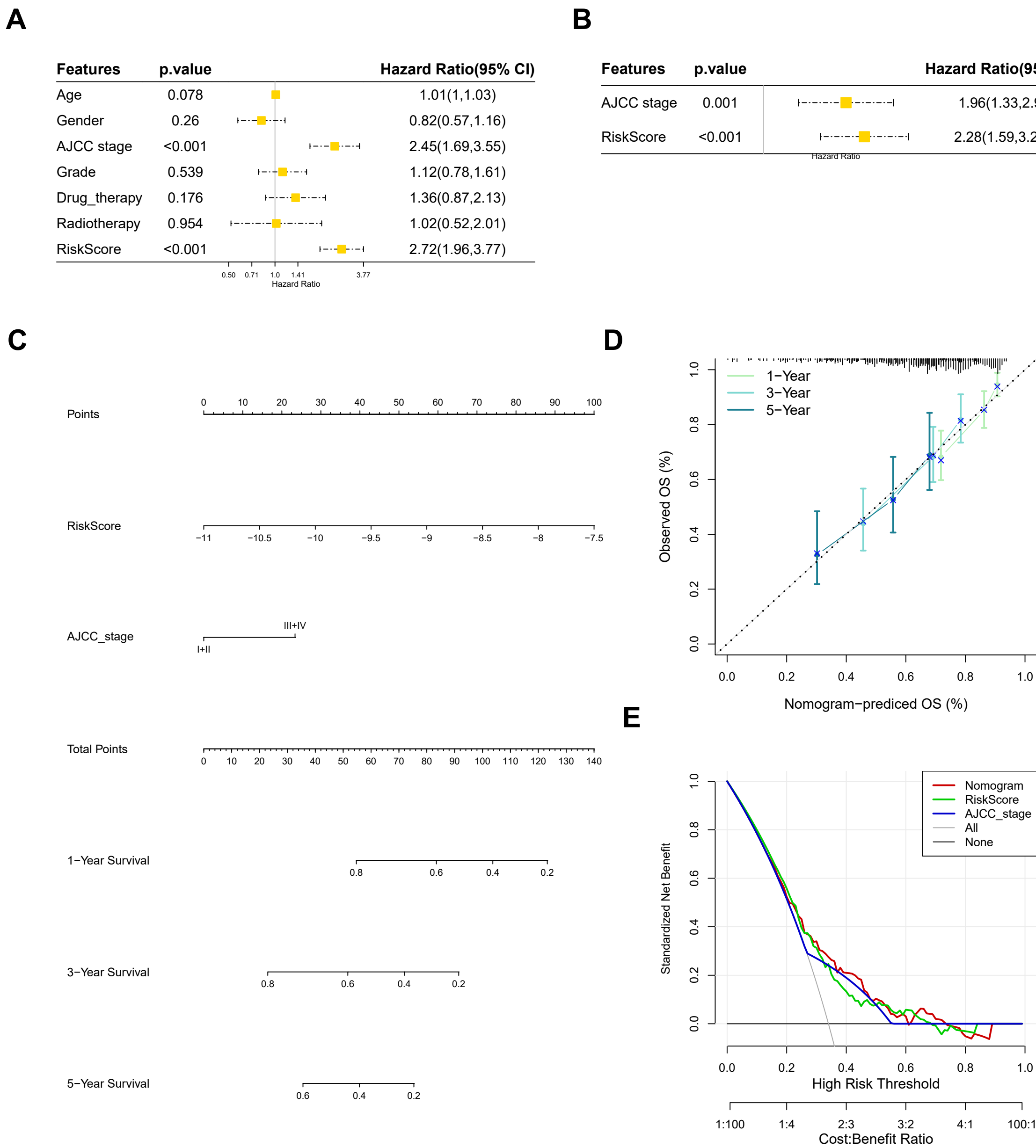

Fig. (S1).

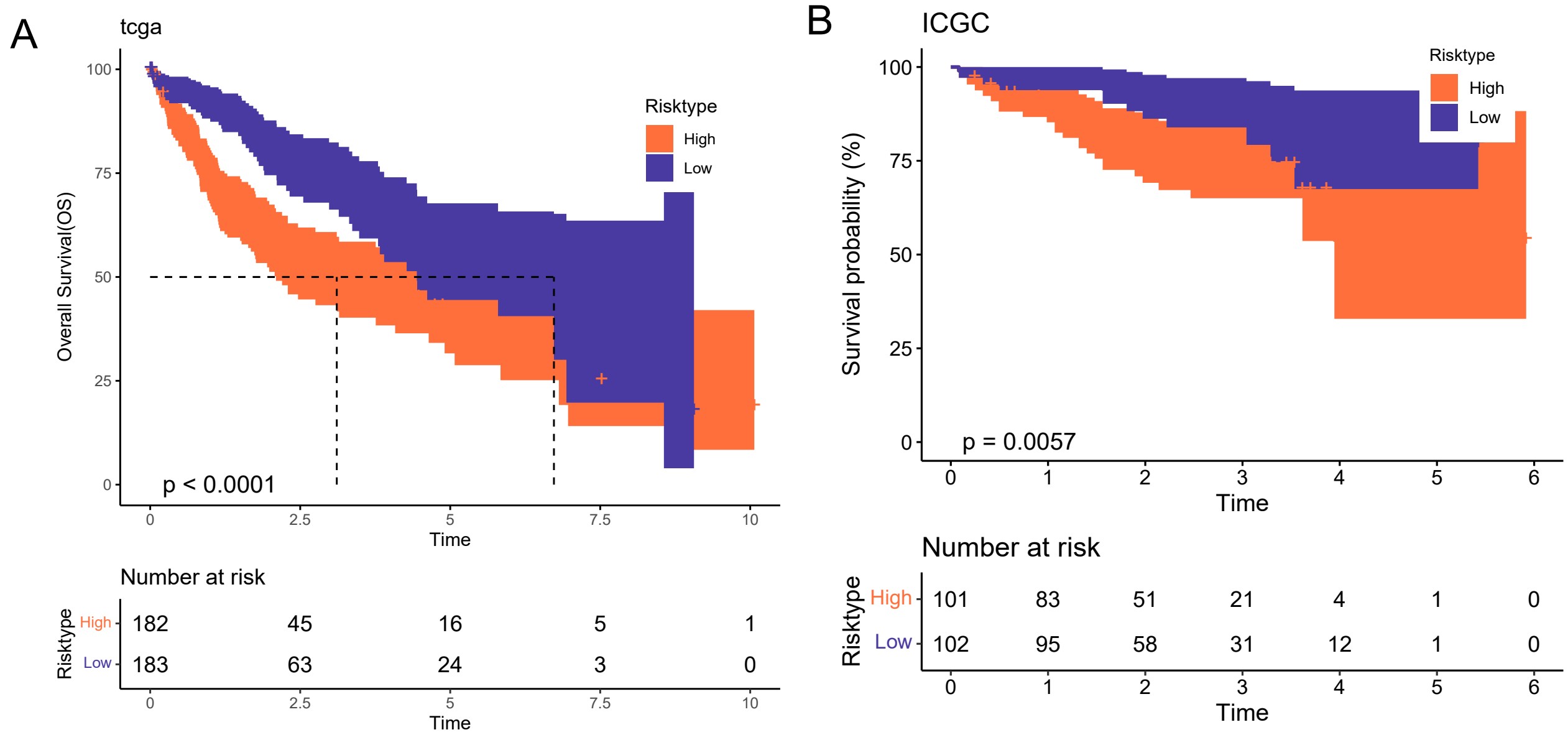
A
tcga
Overall Survival(OS)
100
75
50
25
0
p < 0.0001
Risktype
High
Low
0
2.5
5
7.5
10
Time
Number at risk
Risktype
High 182 45 16 5 1
Low 183 63 24 3 0
B
ICGC
Survival probability (%)
p = 0.0057
Risktype
High
Low
0
1
2
3
4
5
6
Time
Number at risk
High 101 83 51 21 4 1 0
Low 102 95 58 31 12 1 0

Fig. (S2).
